# Perspectives of patients with advanced or metastatic non-small cell lung cancer on symptoms, impacts on daily activities, and thresholds for meaningful change: a qualitative research study

**DOI:** 10.3389/fpsyg.2023.1217793

**Published:** 2023-09-08

**Authors:** Anna Cardellino, Manasee Shah, Jennifer Hanlon, Kimberly Kelly, Alexandra Martin, Aude Roborel de Climens, Sara Taiyari, Alexander Stojadinovic

**Affiliations:** ^1^Patient Centered Outcomes Group, GSK, Collegeville, PA, United States; ^2^GSK, Waltham, MA, United States; ^3^Patient-Centered Solutions, IQVIA, New York, NY, United States; ^4^Patient-Centered Solutions, IQVIA, Paris, France; ^5^GSK, VEO, Stevenage, United Kingdom; ^6^LumaBridge, San Antonio, TX, United States

**Keywords:** patient-reported outcomes, quality of life, advanced non-small cell lung cancer, qualitative research, patient interviews

## Abstract

**Introduction:**

Advanced or metastatic non-small cell lung cancer (NSCLC) is associated with significant symptom burden. It is important to understand the impact of these disease-and treatment-related symptoms on patients’ daily lives and explore from a patient perspective what constitutes a meaningful change in NSCLC symptoms.

**Methods:**

Patient experience of advanced or metastatic NSCLC was explored in this prospective, non-interventional qualitative research study recruiting patients from the United States (US). Interviews were conducted to explore the most important symptoms, daily life impacts, and patients’ perspectives of what constitutes meaningful change when considering their current symptoms versus 6–12 months prior, based on the Patient Global Impression of Severity (PGI-S) and Patient Global Impression of Change (PGI-C) items.

**Results:**

Between February and April 2022, 19 US-based patients with Stage IV NSCLC were recruited; 95% were female, 63% were White, 79% had been diagnosed >1 year prior, and 63% were receiving targeted therapy. Over half the patients indicated their most important symptoms were fatigue, shortness of breath, and cough. Patient differentiation between whether symptoms were disease- or treatment-related lacked concordance, and often patients were unable to distinguish the two. The most frequently mentioned impacts of these symptoms on patients’ daily lives were difficulty walking, sleep disturbance, anxiety/depression, impact on relationships, and difficulty doing daily tasks. Most patients considered a one-point change on the PGI-S or PGI-C to be meaningful based on rating their symptom severity at the time of the interview compared with 6–12 months before the interview.

**Conclusion:**

Based on their own symptom experience, patients with advanced or metastatic NSCLC indicated a one-point threshold for meaningful change, whether improvement or worsening. This suggests a one-point change on the PGI-S or PGI-C may be a potential anchor for patient-reported outcome (PRO) endpoints used in clinical trials. It is important to use PRO instruments that capture the symptoms and impacts identified as most important to patients. These findings highlight the importance of using qualitative methods to assess disease-related symptoms, treatment-related side effects, and the impacts on daily life for patients with advanced or metastatic NSCLC, underscoring how qualitative assessments can complement quantitative PRO instruments for evaluating clinical trials.

## 1. Introduction

Approximately 65% of patients with non-small cell lung cancer (NSCLC) present with advanced or metastatic disease, which is associated with significant symptom burden (Tishelman et al., 2005; Yang et al., 2012; Iyer et al., 2013; Casal-Mouriño et al., 2021). Previous studies have found patients with advanced or metastatic NSCLC report fatigue, shortness of breath, cough, loss of appetite, and pain among their most common disease-related symptoms (Tishelman et al., 2005; Yang et al., 2012; Iyer et al., 2013). Disease-related symptoms are known to worsen as the disease progresses, with an associated negative impact on emotional functioning and health-related quality of life (HRQOL) (Yang et al., 2012; Iyer et al., 2013; Morrison et al., 2017; Teixeira et al., 2022).

Patients with advanced or metastatic NSCLC will likely be on treatment for the rest of their lives, and therefore the impact of both disease-related symptoms and both acute and cumulative treatment-related side effects should be accounted for when evaluating HRQOL (Waisberg et al., 2022). Recommended systemic therapies for patients with advanced or metastatic NSCLC were recently updated to include targeted therapies and immunotherapies (Hanna et al., 2021; Singh et al., 2022). Advocacy groups, medical professional societies, and regulatory agencies have recently pushed for greater monitoring and reporting of HRQOL metrics in clinical trials alongside efficacy and safety measures (Waisberg et al., 2022).

Understanding the patient experience of disease symptoms and treatment-related adverse events (AEs) is vital in improving clinical outcomes and HRQOL, as indicated by regulatory guidance, which recommends inclusion of patient-reported outcome (PRO) instruments to assess disease symptoms and symptomatic AEs in cancer clinical trials (Gnanasakthy et al., 2019). The US Food and Drug Administration (FDA) published guidance in 2009 defining best practices for the development and implementation of PRO instruments and PRO-based endpoints in clinical trials (Food and Drug Administration, 2009) and has since developed guidance documents for patient-centered drug development and conducted a public workshop to provide direction on collecting patient experience data for medical product development and regulatory decision-making (Food and Drug Administration, 2022). In 2021, the FDA released draft guidance defining the core PROs that should be measured in oncology: disease-related symptoms, symptomatic AEs, overall side effect impacts, physical function, and role function (Food and Drug Administration, 2021).

The Patient Global Impression of Severity (PGI-S) and Patient Global Impression of Change (PGI-C) are used to capture symptom severity and change in severity over time, respectively (Guy, 1976). These single item scales are generally used alongside PRO instruments such as the European Organization for Research and Treatment of Cancer-Core Quality of Life Questionnaire (EORTC QLQ-C30), which assesses core cancer symptoms, functioning, and global health status/quality of life. Disease-specific instruments such as the EORTC Quality of Life Questionnaire Lung Cancer 13 (QLQ-LC13) and Non-Small Cell Lung Cancer Symptom Assessment Questionnaire (NSCLC-SAQ) can also be used to evaluate lung cancer symptoms. Although these measures are widely used, the threshold for meaningful change among patients with advanced or metastatic NSCLC using these instruments is not well defined. The FDA has previously remarked on the importance of exploring “whether patients in the target patient population believe that going from ‘very severe’ to ‘severe’ would be considered a ‘meaningful improvement’” when using PGI-S and PGI-C instruments (Food and Drug Administration, 2018). Data are limited on the perspectives of patients with advanced or metastatic NSCLC and thresholds for clinically meaningful change remain an area of unmet need.

The objective of this qualitative interview-based study was to further explore the patient’s experience of advanced or metastatic NSCLC by using open-ended questions to identify the most important symptoms patients report and their impact on patients’ daily lives. The symptoms and impacts identified as important by patients were mapped to concepts included within other commonly used PRO instruments. Additionally, the PGI-S and PGI-C scales were assessed for ease of understanding, and what level of change in PGI-S and PGI-C scales would be considered meaningful to patients based on their current health state and their state 6–12 months before the interview.

## 2. Materials and methods

### 2.1. Study design

This prospective, non-interventional qualitative research recruited patients in the United States (US) for participation in the interview-based study. Recruitment was performed by the third-party firms Just Worldwide and Global Perspectives.

Patients participated in a 45 min web-based interview, facilitated using the Mercuri platform. With patients’ permission, interviews were recorded, and all personal identifying information was redacted from the transcripts before analysis. Interviews conformed to the recommendations of the International Society for Pharmacoeconomics and Outcomes Research (ISPOR) Good Research Practices Task Force (Patrick et al., 2011). One interviewer was used throughout the study and utilized an Institutional Review Board (IRB)-approved semi-structured interview guide containing a set of open-ended questions. The interviewer had prior experience conducting interviews with terminally ill patients and conducted three mock interviews before patient interviews.

### 2.2. Study population

Patients with a confirmed diagnosis of advanced or metastatic NSCLC, defined as American Joint Committee on Cancer TNM Classification of Malignant Tumors (Eighth Edition) Stage IIIa, IIIb, IIIc, and Stage IV, were included in the study (Detterbeck, 2018). Additionally, eligible patients were aged ≥18 years; self-certified as having experienced a change in disease state (e.g., symptom severity, frequency, or interference with daily living) within the past 6–12 months; able to speak, write, and read English fluently; willing and able to participate in a 45 min interview; residents of the US; and willing and able to provide informed consent. Patients were excluded if they had symptomatic brain metastasis; had taken >20 mg of corticosteroids per day in the last 6 months; had a chronic mental health illness that would prevent them from completing a 45 min interview (as confirmed by physician/physician’s office staff); had been diagnosed with a separate primary cancer within 2 years of the interview date; or had a positive COVID-19 test in the last 6 months.

### 2.3. Interview structure

#### 2.3.1. Concept elicitation

Before their interview, patients were advised to reflect on their recent experience (past 6–12 months) with NSCLC, including how their symptoms have changed and how these changes have impacted their life. This preparation allowed the interviewer to focus the discussion quickly and accurately on temporally relevant symptoms and impacts, as well as confirm that the patient had experienced a recent change in disease state (per the study population criteria above). In the first part of the interview, patients were asked to spontaneously describe in their own words their symptomatology; specifically, their three most important symptoms and the impact of those symptoms on their daily life.

#### 2.3.2. Cognitive debriefing

The second part of the interview assessed patients’ understanding of the PGI-S and PGI-C scales. The PGI-S asked patients to report the overall severity of their cancer symptoms over the past 7 days using a five-point Likert scale with the response options “no symptoms,” “mild,” “moderate,” “severe,” and “very severe.” Changes in patients’ cancer symptoms were reported via the PGI-C, where patients were asked about their current symptoms compared with their experience 6–12 months before the interview. Change was reported via a five-point Likert scale with the response options “much better,” “a little better,” “no change,” “a little worse,” and “much worse.” The PGI-S and PGI-C items were shared with patients through an online screen-sharing program, and patients were asked to respond to the questions based on the symptoms reported in the concept elicitation part of the interview. Patient understanding of these measures was assessed through a cognitive debriefing interview, which included questions focused on the clarity and understandability of the PGI-S and PGI-C; relevance to patients’ experience with NSCLC; and the clarity, understandability, and appropriateness of response options.

#### 2.3.3. Meaningful change

Finally, patients were asked to describe what would constitute a meaningful improvement and worsening of their most important symptoms and the impact of those changes on their daily lives. Patients were asked to define their responses to the PGI-S in their own words. For example, if a patient reported “moderate” fatigue, they were asked to describe what this symptom and severity level meant to them. Patients were then asked to consider their symptoms 6–12 months prior and report their symptom severity on the PGI-S. Considering this real-life symptom change as a reference, patients were asked to describe what would constitute a meaningful improvement and worsening of their symptoms on the PGI-S. The interviewer asked open-ended and probing questions to determine the smallest change the patient would deem meaningful and how that change may impact their daily life. When reporting meaningful change, it was important that patients considered the recent (past 7 days vs. 6–12 months prior) change experienced in their symptomatology to ensure reported perspectives were relevant and accurate.

In a similar way, using their symptom change previously reported via the PGI-C, patients were asked to consider the smallest improvement or deterioration that they would consider meaningful on the PGI-C item.

### 2.4. Study conduct, management, and ethics

The study protocol was reviewed and approved by the WIRB-Copernicus Group, Inc. IRB (Review #: 20216504) before any study activities began. The study was conducted in accordance with the protocol, IRB requirements, applicable regulations and guidelines, and ethical principles for the conduct of research. Upon completion of the study interview, patients were thanked for their time and received an honorarium payment for their contributions, which was facilitated by Just Worldwide and Global Perspectives.

### 2.5. Data analysis

De-identified transcripts of patient interviews were coded using a qualitative research software (MAXQDA). A codebook was developed that evolved as new concepts emerged from interviews. Two coders were involved in the study: a primary coder and a senior-level researcher. Both coders coded the first transcript collaboratively to establish the coding process. The second transcript was coded by both coders independently, and an acceptable inter-coder agreement (ICA), pre-defined as Krippendorff’s C-alpha binary >0.7, was met. The primary coder conducted the coding of remaining transcripts, with weekly alignment meetings with the senior-level researcher to allow for discussion and alignment on sections of text that were difficult to code, and iterative updates to the codebook.

Data regarding patients’ symptoms, the impact of symptoms on daily life, and meaningful change were summarized using descriptive statistics. If a patient reported a PGI-S or PGI-C response between two ratings, the more severe rating was coded for the purposes of analysis (e.g., a “severe or very severe” response was coded as “very severe”). If a patient indicated a response outside the response options (e.g., “better than much better”), the nearest available response (e.g., “much better”) was recorded.

## 3. Results

### 3.1. Study population

A total of 19 patients were interviewed between February 2022 and April 2022. Baseline demographics are presented in Table 1. All participants were residents of the US. The mean age was 52 years (range 28–71). Eighteen patients were female (95%), and 12 patients (63%) were White. Most patients were at least college educated (*n* = 13, 68%), and six (32%) were employed full- or part-time. All patients had Stage IV NSCLC, and 15 (79%) had been diagnosed longer than 12 months before the study, three of whom were diagnosed more than 6 years before the study. Most patients were receiving second-line or third-line therapy (both *n* = 6, 32%); however, three patients (16%) were not receiving any treatment. Of the 16 patients receiving treatment, 12 (63%) were receiving biomarker- or mutation-targeted therapy (excluding immunotherapy).

**Table 1:** Baseline demographics and clinical characteristics.

	Total number of patients, *n* (%) (*N* = 19)
*Age in years*	
25–40	4 (21)
41–55	8 (42)
56–69	5 (26)
≥70	2 (11)
*Sex*	
Female	18 (95)
Male	1 (5)
*Race*	
White	12 (63)
Other	3 (16)
Black/African American	2 (11)
Asian	2 (11)
*Ethnicity*	
Not Hispanic or Latino or Spanish origin	18 (95)
Hispanic or Latino or Spanish origin	1 (5)
*Education level*	
High school	2 (11)
Associate degree	4 (21)
College/some college	10 (53)
Graduate	3 (16)
*Employment status*	
Full-time	3 (16)
Part-time	3 (16)
Retired	4 (21)
Homemaker	3 (16)
Unemployed	3 (16)
Disabled	3 (16)
*Time since initial NSCLC diagnosis* ^a^	
<1 year	4 (21)
1–3 years	5 (26)
4–6 years	7 (37)
>6 years	3 (16)
*Current treatment line*	
No treatment	3 (16)
1st line	1 (5)
2nd line	6 (32)
3rd line	6 (32)
4th line	2 (10)
5th line	1 (5)
*Current treatment type*	
Targeted therapy	12 (63)
No treatment	3 (16)
Chemotherapy	2 (11)
Immunotherapy	1 (5)
Immunotherapy + chemotherapy	1 (5)

a Please note that time since diagnosis may not represent time since diagnosis of Stage IV disease.

### 3.2. Symptoms and impacts

**Figure 1:**
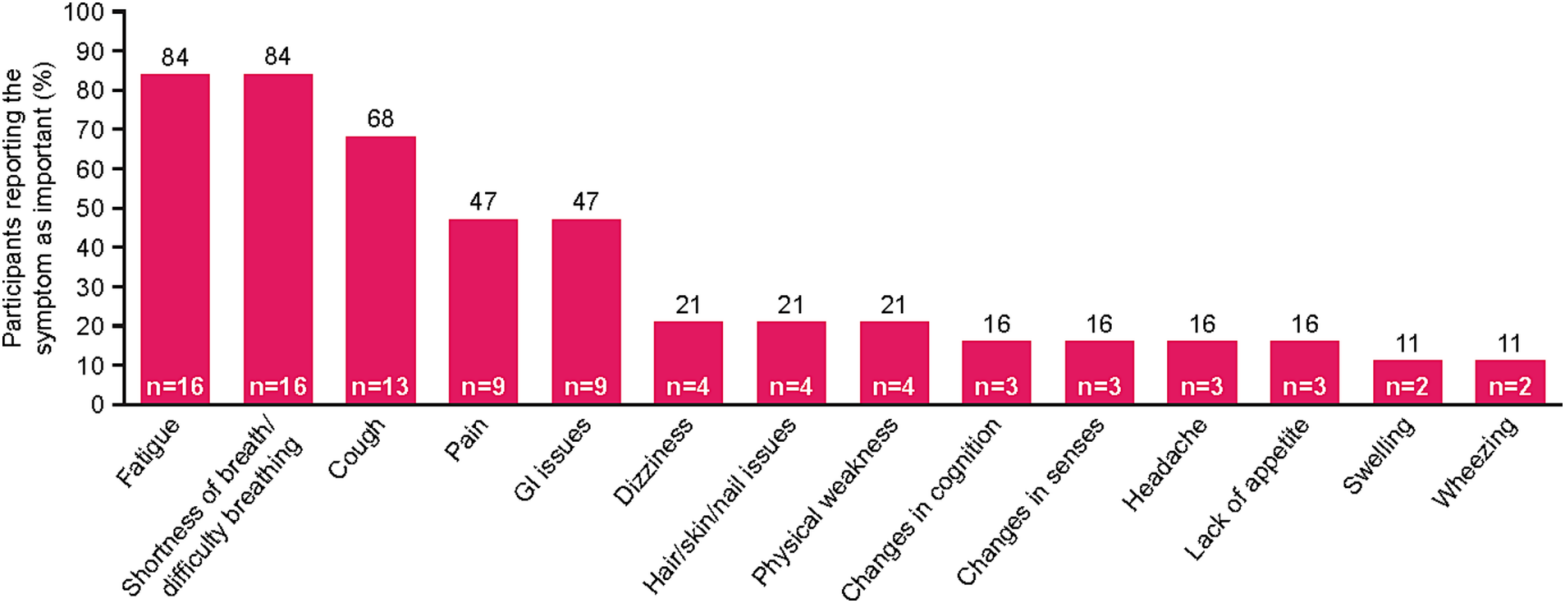
NSCLC-specific symptoms mentioned by patients during interview. Patients were asked to list their three most important symptoms. Two patients reported “no symptoms.” GI, gastrointestinal; NSCLC, non-small cell lung cancer.

Fourteen unique symptoms were reported by patients as the “most important symptoms” throughout their NSCLC disease journey (Figure 1). More than half of the recruited patients reported fatigue (*n* = 16, 84%), shortness of breath/difficulty breathing (*n* = 16, 84%), and/or cough (*n* = 13, 68%). Patients also commonly reported pain (*n* = 9, 47%) and gastrointestinal (GI) issues (*n* = 9, 47%). Patient quotes describing these spontaneously reported symptoms are presented in Table 2.

**Table 2:** Patient quotes describing most commonly reported disease-related NSCLC-specific symptoms.

Symptom (mentions)	Quote
Fatigue (*n* = 16)	“Lately I’ve been feeling really fatigued. I really do… I get that way and I just have to lie down. I cannot even read a book or watch TV. I really have to close my eyes and just rest.”
	“There would be like times where in the middle of the day I feel like I cannot even open my eyes at all, and I just have to take a nap. I’m not usually one that naps very much.”
	“The fatigue is consistently present.”
Shortness of breath/difficulty breathing (*n* = 16)	“I would sit over my legs, like bend over my legs because that made it kind of easier to breathe. I did not realize how bad I was. I was really, really bad.”
	“The shortness of breath, well checking pulse ox, which I guess would go under shortness of breath. That’s the main one for me.”
	Interviewer: “I’m curious if you can talk to me a little bit about what you think are the three most important symptoms that you have experienced as a result of your lung cancer?” Patient: “Definitely shortness of breath.”
Cough (*n* = 13)	“It (the cough) was dry, nonproductive, constant.”
	“A cough, number one, and it’s not just like a cold cough, it’s like a cough.”
	“I think the coughing is involuntary. I do not know when I’m going to cough. Yeah, I wasn’t thinking about it.”
Pain (e.g., chest, back; *n* = 9)	“I was experiencing discomfort in my back. There was pain when I tried to sneeze or when I tried to do deep inhalation. It got worse really fast…”
	“Back pain… At some point the back pain was unbearable…”
	“A substitute doctor was there, and I think she figured it out. I was having shortness of breath, chest pain.”

All patients reporting GI issues described these as treatment-related, and therefore quotes on this symptom are not included. GI, gastrointestinal; NSCLC, non-small cell lung cancer.

For some patients, defining symptoms as disease-related or treatment-related was challenging. Of the patients who reported feeling fatigue (*n* = 16), nine (56%) identified fatigue as disease-related, four (25%) identified it as treatment-related, and three (19%) had difficulty defining it as disease-related or treatment-related. Mentions of shortness of breath/difficulty breathing (*n* = 16) were most often categorized as disease-related (*n* = 12, 75%), with only one patient reporting this symptom as treatment-related (6%) and three patients unable to distinguish (19%). Similarly, most patients who mentioned pain (*n* = 9) defined it as disease-related (*n* = 6, 67%), whereas one patient defined it as treatment-related (11%), and two were unable to distinguish the origin (22%).

GI issues, hair/skin/nail effects, changes in senses (e.g., vision/taste), and headache were identified as treatment-related by all patients who reported them.

**Figure 2:**
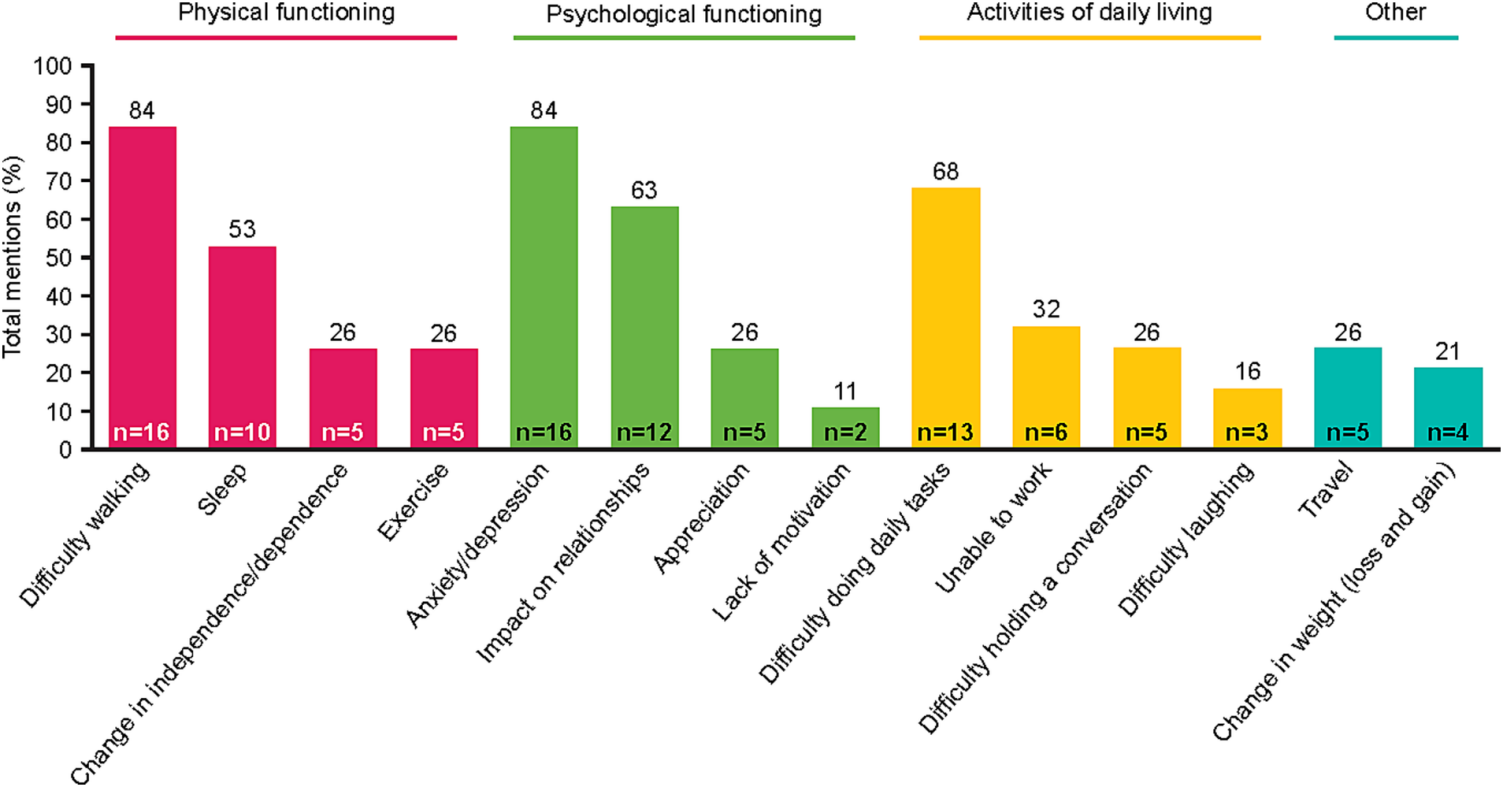
Impact of symptoms mentioned by patients during interview.

To understand the patient experience of NSCLC, impacts of disease-related and treatment-related symptoms were captured. Fourteen impact-related concepts were identified by patients spanning three main domains: physical functioning, psychological functioning, and activities of daily living (Figure 2). Across these domains, five impacts were described by ≥50% of patients: difficulty walking, sleep disturbance, anxiety/depression, impact on relationships, and/or difficulty doing daily tasks. Notably, difficulty walking was reported by many patients as being greatly affected by shortness of breath and fatigue. Difficulty walking limited many patients’ ability to traverse their own homes. Often, patients linked their anxiety to an overwhelming sense that their disease was going to get worse, particularly during periods when their symptoms were minimal. Patients’ ability to perform some daily tasks (including cooking, cleaning, laundry, grocery shopping, etc.) was impacted by multiple NSCLC symptoms, including shortness of breath, fatigue, and pain. Some patients also reported muscle atrophy and weakness due to an inability to exercise; this contributed to their difficulty performing tasks of daily living. Patient quotes describing how some symptoms and impacts were linked are presented in Table 3.

**Table 3:** Patient quotes linking specific symptoms to impacts on activities of daily living.

Impact (mentions)	Quote
Difficulty walking (*n* = 16)	“At one point, I had difficulty breathing or doing light… or just walking around the house or trying to get something from the cupboards… so difficulty breathing when I’m taking short walks. I would have to catch my breath.”
	“Okay. I enjoy walking, considering that’s probably the only exercise I get. I live in an apartment complex where it’s all indoors. To get mail or packages, I mean, I have to walk down a couple long hallways. Then there’s an elevator. In the past, it was my daily exercise. It was no problem at all. I did not have a problem with breathing or anything. Whereas now all of a sudden…I’m finding that as I’m walking, I have to stop for a while and release the mask and breathe.”
	“I get very short of breath walking just a short distance. Walking up and down steps is very difficult.”
Anxiety/depression (*n* = 16)	“The anxiety of pain. I have a…I mean, well, most people do…have a thing against having any kind of pain. Lung cancer never… Lung cancer itself never caused me pain. The treatments of the lung cancer cause me the pain. That’s the fear of…the fear of pain. Nobody wants it.”
	“(The) cough and the back pain. Those are the two that are very… I want to say triggering… For cancer patients, if you cough… my immediate thought is, ‘Hey, it’s back.’ Right now, I’m… you know, I have progression again. I want to say that the cough and the back pain are the two (most important symptoms) for me, personally.”
	“Not being able to get your breath is the most frightening thing. It’s like you are being suffocated, right, but you are watching TV.”
Difficulty doing daily tasks (*n* = 13)	“You cannot really do your daily tasks when you have shortness of breath… The most bothersome was definitely the shortness of breath. Because sometimes even going from my bed to the bathroom would leave me out of breath.”
	“When I’m in the garden, I do stuff and then I have to just stop doing what I’m doing and sit down for a little bit because I’m feeling it. Then a few minutes later, I go back to doing it or I just stop doing it and do some other stuff. I can still do the same things, it’s just that I’m limited.”
	“Another example would be like I go to the same grocery store for both gasoline, pharmacy, and food. If I have to do all of those in one trip, then I’m on my feet a lot longer and that’s really going to hurt my back. It’s severe while I’m doing that. So, then I come home and I just lay flat out on my back and then that, after a couple of hours, brings it back down to the moderate level.”

### 3.3. Concept coverage in EORTC PRO instruments

As an additional analysis, the concepts (most important symptoms and impacts on daily life) spontaneously mentioned by patients in their interviews were mapped to the existing PRO instruments commonly used in NSCLC: EORTC-QLQ-C30, EORTC-QLQ-LC13, and NSCLC-SAQ (Supplementary Table S1). The EORTC-QLQ-C30 included 50% (7/14) of the symptoms identified by patients within this study, whereas symptom coverage of the EORTC-QLQ-LC13 and NSCLC-SAQ was less at 21% (3/14) and 36% (5/14), respectively. Impact coverage was 57% (8/14) for the EORTC-QLQ-C30 and not relevant for the EORTC-QLQ-LC13 nor the NSCLC-SAQ focusing on NSCLC symptoms only. In terms of the most important symptoms, shortness of breath and pain were covered by all three instruments, with fatigue and lack of appetite covered only by EORTC-QLQ-C30 and NSCLC-SAQ. As may be expected for a tumor-agnostic instrument, cough was not mentioned in the EORTC-QLQ-C30 but was mentioned in both NSCLC-specific instruments. The following symptoms identified by patients within this study were not covered by any of the PRO measures: dizziness, hair/skin/nail changes, changes in senses, headache, swelling, and wheezing. The following impacts identified by patients within this study were not covered by any of the PRO measures: exercise, appreciation, lack of motivation, difficulty holding a conversation, difficulty laughing, and change in weight.

### 3.4. Cognitive debriefing

All patients found the PGI-S and PGI-C items and response options easy to understand, as evidenced by their accurate rephrasing of the questions in their own words when prompted. However, three patients did provide unsolicited feedback on the PGI-S, commenting upon the queries’ broad scope and short recall period, and four patients recommended more options and a longer recall period. For the PGI-C, three patients recommended clarifying “study” was intended to refer to a “clinical trial,” while eight patients recommended revisions to the response options (*n* = 2, include numerical scale; *n* = 4, further clarify response options to reduce generalization of symptoms and subjective nature of severity quantification; *n* = 2, add more intermediate response options).

### 3.5. Meaningful change: PGI-S item

When asked to assess the severity of their cancer symptoms over the last 7 days per PGI-S criteria, most patients selected “mild” or “moderate” (each *n* = 7, 37%). The interpretation of “mild” was that patients might notice the symptoms, but they were still able to live a generally normal life, while “moderate” symptoms were not severe enough to be alarming, but they remarkably impacted patients’ daily lives more than “mild” symptoms. Responses of “severe” (*n* = 2, 11%), “very severe” (*n* = 1, 5%), and “no symptoms” (*n* = 2, 11%) were less common. By comparison, response options were more heterogeneous but broadly more severe when rating symptoms experienced 6–12 months before the interview, with “moderate” or “severe” (each *n* = 5, 26%) followed by “no symptoms” (*n* = 4, 21%), “very severe” (*n* = 3, 16%), and lastly “mild” (*n* = 2, 11%). Patients’ interpretation of “severe” included needing medications to relieve their symptoms such as pain and anxiety, and to help them sleep. Patients who described their symptoms as “very severe” expressed their symptoms to be so bad that it would be difficult to understand or imagine for someone without the disease.

**Figure 3:**
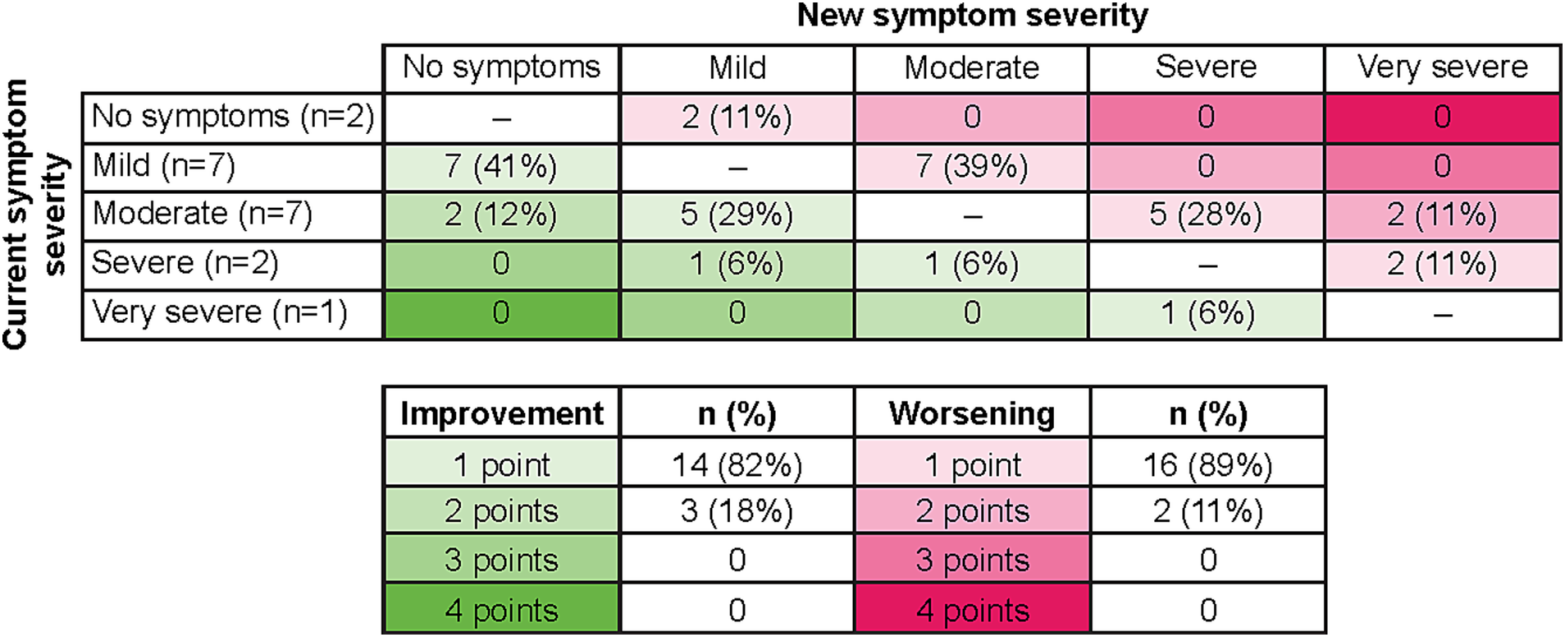
Relationship of current symptoms to perception of meaningful improvement and worsening on PGI-S. As two patients selected “no symptoms” to describe their last 7 days, no improvement options were available, and therefore *n* = 17 for improvement. As one patient selected “very severe” to describe their last 7 days, no worsening options were available, and therefore *n* = 18 for worsening. PGI-S, Patient Global Impression of Severity.

Seventeen patients were able to indicate what they would consider meaningful improvement in their cancer symptoms (excluding *n* = 2 who reported “no symptoms”), and 18 indicated what they would consider meaningful worsening (excluding *n* = 1 who reported “very severe”) using the PGI-S scale. Most patients identified a one-point improvement on the PGI-S scale to be meaningful (82%, *n* = 14), whereas some believed a two-point change was needed (18%, *n* = 3; Figure 3). This may be partially due to their current symptom severity, as patients who indicated a two-point change was needed to be considered meaningful included two patients who rated their symptoms “moderate” and one patient who rated their symptoms “severe,” with a patient stating, “It (a one-point change) would not be significant enough for me…. It would be better, but not significant… To me the word significant means a lot.” Similarly, most patients identified a one-point worsening to be meaningful (89%, *n* = 16), whereas some believed a two-point worsening was required (11%, *n* = 2; Figure 3). Quotes illustrating the patient perspective on meaningful change in the context of specific symptoms or their full symptomatology are presented in Table 4. Several patients expressed doubt that they would be able to achieve “no symptoms” at any time, particularly those who reported “mild” symptoms in the last 7 days.

**Table 4:** Quotes describing patients’ perspectives on meaningful change per PGI-S.

Point-change deemed meaningful (mentions)	Quote
One-point improvement (*n* = 14)	*Fatigue; change from* “*mild*” *to* “*no symptoms*”: “I would go back to prior to being diagnosed. I do not feel tired. I could go to 12 h workdays. My previous job made me do a lot of different field calls, so I hope to go back to that. There would be no symptoms for me.”
	*Full symptomatology; change from* “*moderate*” *to* “*mild*”: Interviewer: “If you are here today at ‘moderate,’ where would you want to move on the scale for you to consider it to be a significant or important improvement?” Patient: “Mild.”
	*Physical weakness; change from* “*moderate*” *to* “*mild*”: “‘Moderate’ I would still need resting periods of weak and breathing problems. ‘Mild’ is just less where I feel it but it’s not affecting my day or making… dictating what I do with my life.”
	*Shortness of breath/difficulty breathing; change from* “*severe*” *to* “*moderate*”: “So, a meaningful change for me at this point would be ‘moderate’ because it would be at least… It would have those times of manageability where I wasn’t so affected by my symptoms… I’d say just having energy, fatigue, tiredness, shortness of breath. Just an overall feeling of just feeling not well.”
	*Fatigue; change from* “*very severe*” *to* “*severe*”: “When my fatigue was ‘severe,’ I could work through it, you know, go get the mail, maybe sort through it, and stuff like that. Maybe walk a half a block or a block. Now (at ‘very severe’), I could not. It would be really difficult to walk a block.”
Two-point improvement (*n* = 3)	*Shortness of breath/difficulty breathing; change from* “*severe*” *to* “*mild*”: “From ‘severe’ to ‘mild’ (would be meaningful) because that’s when I could still do quite a bit of everything and maybe be bothered by the symptom but not affected.”
	*Shortness of breath; change from* “*moderate*” *to* “*no symptoms*”: “The difference? (At ‘mild’) you still have the symptom, so whatever your problem was, so let us call mine shortness of breath. Okay? I still have my shortness of breath. It’s maybe not as often, maybe I can walk 50 feet instead of being able to walk 100 feet. That’s the difference… Maybe that’s the way of putting it. I want to be able to walk 100 feet without even having to… I do not want to have to think about it.”
One-point worsening (*n* = 16)	*Pain and cough; change from* “*no symptoms*” *to* “*mild*”: “I’m very sensitive to pain and things in my body, so (‘mild’). I know that something is wrong, that the (‘mild’) would mean a very mild pain in my back with breathing… with deep breathing, yawning, and hiccups… If my spot in my back hurts with the hiccups, with a yawn, with a deep breath, that to me is a ‘mild’… More frequent coughing. So, all this means that I have… my cancer treatment is not working anymore… If I cough now… if I start coughing, that would be extremely meaningful to me.”
	*Shortness of breath/difficulty breathing; change from* “*mild*” *to* “*moderate*”: “When I start feeling short of breath again… when I start feeling… and then the sats start going down where I start feeling like, ‘Uh-oh, I need oxygen again,’ that’s a problem.”
	*Pain; change from* “*moderate*” *to* “*severe*”: “(At ‘moderate’) I move around… Like right now, I have pain. It’s there. I know when I change positions, then it’s going to relieve that pain. As opposed to when I was at ‘severe’, nothing changed it (the pain) no matter what I did. Nothing changed it; nothing relieved it.”
Two-point worsening (*n* = 2)	*Fatigue; change from* “*moderate*” *to* “*very severe*”: “‘Severe’ would be… I would actually go to the car to go shopping, park the car at the supermarket, get out of the car, then say, ‘You know, I just do not feel up to it’ and get back in the car. Where ‘very severe’ would be I would never even get to the car. I’d just stay home in the first place and just not get out of my pajamas and say, ‘You know, I just cannot go out. I just cannot do it.’”

PGI-S, Patient Global Impression of Severity.

### 3.6. Meaningful change: PGI-C scale

**Figure 4:**
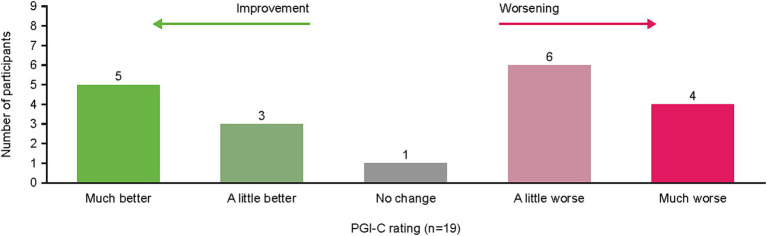
Patient PGI-C rating of symptom change from “previous” (6–12 months ago) to “current” (last 7 days) during interview. PGI-C, Patient Global Impression of Change.

When asked to rate their change in cancer symptoms in the last 7 days compared with the prior 6–12 months per the PGI-C, the option “a little worse” was reported by 32% (*n* = 6) of patients and “much better” by 26% (*n* = 5) of patients (Figure 4). Illustrative patient quotes are presented in Table 5. Patients’ interpretations of “much better” included absence of symptoms or symptoms no longer interfering with daily living; “a little worse” was used by patients whose symptoms were currently more bothersome than previously but remained manageable. In contrast, “much worse” was chosen by patients whose daily lives had been significantly affected and had seen noticeable deterioration in their symptoms. Notably, only one patient reported “no change” in symptoms and clarified they would have chosen “not applicable” if that were an option, since they lacked disease symptoms during or following treatment.

**Table 5:** Quotes describing patients’ perspectives of their response option selection on PGI-C.

Response option (mentions)	Quote
Much better (*n* = 5)	“My change in my symptoms would be ‘much better’… Just because I do not really feel like I have any symptoms right now from the lung cancer. Yeah, I mean, I went from having symptoms to basically not having any symptoms.”
	“(‘Much better’ means) a dramatic change in your symptoms compared to how it was six months ago, or when you started your treatment”
	“It’s like night and day how much better I am doing.”
A little better (*n* = 3)	“It is only ‘a little better,’ only because the intensity is not as much as last year, but it’s still there.”
No change (*n* = 1)	“I did not have noticeable symptoms from the disease itself when I was in treatment… This is asking about the disease symptoms, where I did not have that, so, yeah, ‘no change,’ or, yeah, ‘none.’ Yeah, ‘not applicable.’”
A little worse (*n* = 6)	“I would say ‘a little worse’… I’ve noticed the symptoms getting worse. I hesitate to characterize it as ‘much worse’ because it’s still manageable at home. It’s not often. It’s not a cause for medical intervention, really, yet.”
	“It went from no symptoms to having some symptoms. That, obviously, is ‘a little worse’ because the goal is ‘no symptoms.’”
	“I would say I’m ‘a little worse.’ I would not say ‘much worse’ because I think that’s a little bit more severe than what I’m experiencing. So, I would definitely say I’m feeling ‘a little worse’ than a year ago. I think if I was to continue to feel more symptoms, worsening symptoms, it would… definitely my answer would be ‘much worse.’”
Much worse (*n* = 4)	“Thinking back the individual 10 months (…) I was, of course, ‘much worse.’ I was feeling terrible and I’m still feeling terrible. It’s a constant progression of worsening.”
	“Today, I’m ‘much worse’ than I was back when I first got diagnosed (last year) … Noticeable, very noticeable change.”

PGI-C, Patient Global Impression of Change.

Patients were also asked whether they would consider a response of “no change” on the PGI-C to be a significant or important outcome, of which 10 of 18 (56%) responded “yes” and the other eight (44%) “no.” Interestingly, regardless of whether patients reported the outcome was meaningful, patients in both response groups disagreed about whether this would be a positive or negative outcome. Some respondents stated “no change” represented absence of progression, which was viewed as positive, although the significance of this finding varied. For example, one patient stated, “no change is stable and stable with my kind of cancer is really good”; however, this patient went on to say they did not view this as a significant outcome. In contrast, other respondents said “no change” may indicate the treatment was ineffective and could cause them to explore alternative treatment options, which some reported as an important finding whereas others viewed it as inconclusive. For example, one patient stated, “if it were ‘no change’ and I had been having severe symptoms, then that would definitely be significant and concerning and make me wonder whether or not the treatments were working.” Another patient suggested their impression of “no change” depended on their state when beginning treatment: “if I could bottle up where I was in June and keep it there and maintain, that would be, I could tolerate that. (…) I do not want to maintain at (my current) level because this is miserable. I might as well not take (anything).”

When asked what would constitute a meaningful symptom improvement on the PGI-C scale, most participants indicated a one-point improvement (*n* = 17/19), with a two-point improvement the only other chosen answer (*n* = 2/19). This same distribution was also observed for patients’ assessments of meaningful worsening.

Patient perspective of what constitutes a meaningful symptom change by PGI-S and PGI-C scales was stratified by time from NSCLC diagnosis (Supplementary Table S2). Most patients considered a one-point symptom change (improvement or worsening) meaningful regardless of whether they received their diagnosis within the last year or had been diagnosed for longer than 1 year. This was consistent across both PGI-S and PGI-C.

## 4. Discussion

This prospective, interview-based qualitative research study of 19 patients in the US who had been diagnosed with Stage IV NSCLC identified the most important symptoms and impacts spontaneously reported by patients and what constituted a meaningful change in their symptoms based on the PGI-S and PGI-C.

A variety of symptoms were reported by patients, which were considered disease-related, treatment-related, or of indefinite origin. Almost half of patients reported the same five symptoms (fatigue, shortness of breath/difficulty breathing, cough, pain, and GI issues) consistently. Of these, the top three disease-related effects (fatigue, shortness of breath/difficulty breathing, and cough) were in alignment with those reported in the literature (Yount et al., 2012; Williams et al., 2022). For fatigue, shortness of breath, pain, dizziness, physical weakness, and swelling, up to three patients per symptom had difficulty defining whether the cause was NSCLC or their treatment. Similarly, most symptoms had a mixture of patients reporting them as singularly disease- or treatment-related. In sum, these results suggest that treatments for NSCLC can cause side effects that overlap with or exacerbate disease-related symptoms, and some patients may be unsure which symptoms are caused by their disease versus their treatment. In terms of clinical management, this overlap of disease- and treatment-related symptoms may make it challenging to identify their origin and therefore the appropriate management strategy (e.g., dose reduction for treatment-related nausea versus antiemetic use for disease-related nausea). Furthermore, this ambiguity reinforces the need to evaluate both disease- and treatment-related symptoms in PRO scales used in clinical trials.

The list of spontaneously reported symptoms was further elaborated when patients discussed how the symptoms impact their lives. Reported impacts mapped to three domains: physical functioning, psychological functioning, and activities of daily living. Across these domains, difficulty walking, anxiety/depression, impact on relationships, and difficulty doing daily tasks were identified as the most impactful for patients’ lives. Here, the qualitative methodology of this study collected patient quotes that helped illustrate the relationship between symptoms and impacts, clarifying which symptoms most heavily contributed to each impact. For example, difficulty walking was often attributed to fatigue and less often to muscle weakness; impact on social/interpersonal relationships was often due to patients lacking the energy to maintain those relationships.

As an additional analysis, we reviewed PRO scales commonly used in clinical trials and identified key similarities and differences between the outcomes evaluated in each scale and the symptoms and impacts on daily living spontaneously reported by patients in our study. We found wide variation between instruments in concept coverage in the EORTC-QLQ-C30, EORTC-QLQ-LC13, and NSCLC-SAQ. Overall, half of all unique symptoms identified were covered by at least one questionnaire, and all of the most frequently reported symptoms (fatigue, shortness of breath/difficulty breathing, cough, pain, and GI issues) were covered by either the EORTC-QLQ-C30 or EORTC-QLQ-LC13. Cough is included within disease-specific EORTC-QLQ-LC13 and NSCLC-SAQ, but not EORTC-QLQ-C30. Six of the important symptoms spontaneously reported by study participants were likely treatment-related and were not covered by any of the three scales. The disease-specific questionnaires (EORTC QLQ-LC13 and NSCLC-SAQ) do not cover impacts; therefore, impacts are assessed only by EORTC-QLQ-C30. EORTC-QLQ-C30 included over half the impacts identified by patients including all the most frequently reported impacts, but did not include exercise, appreciation (e.g., for life, people, time), lack of motivation, difficulty holding a conversation, difficulty laughing, and change in weight (loss or gain). To ensure a patient-centered trial design, clinical trials evaluating treatments for advanced or metastatic NSCLC should prioritize measuring symptoms identified by patients as the most important, particularly fatigue, dyspnea, cough, pain, and GI issues. Taken together, these findings support using EORTC-QLQ-C30 in conjunction with a disease-specific PRO measure for comprehensive symptom and impact coverage. Considering the inclusion of a PRO treatment side effect measure (e.g., PRO-Common Terminology Criteria for Adverse Events) could account for those symptoms identified as important and not covered by the three evaluated measures (Smith et al., 2016). Furthermore, the results emphasize the benefits of using qualitative interview-based methods to identify symptoms and impacts that are most important to patients but not covered by commonly used PRO instruments.

Importantly, the methodology of this study requested patients evaluate what constitutes meaningful change in their symptoms qualitatively and based on their real-world experience. This experience-based (vs. a hypothetical scenario-based) approach allowed patients to better articulate their interpretation of a one-point improvement or worsening, based on their own history. For some patients with “mild” symptoms in the last 7 days, they remarked that a one-point improvement to “no symptoms” may be unrealistic or unachievable. This may suggest there could be an additional clinically meaningful category between “mild” and “no symptoms” in which symptoms are still present but do not interfere with activities of daily living. Further, many patients described meaningful change in terms of impact on their daily lives in addition to or instead of specific symptoms highlighting the importance of evaluating both impacts on daily activities and symptom severity to capture the full scope of HRQOL impacts. Variation in the symptom change deemed as clinically meaningful by patients may reflect the diversity in treatment lines being received by patients, which ranged from first-line setting through to fifth-line setting, as well as including some patients who were receiving no therapy.

Patients found both the PGI-S and PGI-C scales easy to understand and largely suitable for use for other patients with NSCLC. In terms of identifying what would constitute a meaningful change on either scale, most patients reported a one-point change in either direction (improvement or worsening) to be meaningful. We had hypothesized that patients with longer durations since diagnosis would be more tolerant of a one-point change than patients who had been diagnosed more recently, possibly due to lower expectations. The results of this study do not support this idea as the four patients who had been diagnosed for ≤1 year also tended to consider one-point changes meaningful. However, this study had a small sample size and further research should be conducted to further elucidate the relationship between time since diagnosis and definition of meaningful change.

The strength of this study lies in its qualitative collection of perspectives from patients with advanced or metastatic NSCLC, using their prior experience with disease (6–12 months prior) compared with their current state (last 7 days). This contrasts with discrete-choice experiments (Duong et al., 2021; Raymond et al., 2021), which, while powerful, focus on hypotheticals and may fail to observe underlying relationships between baseline characteristics, symptom severity, and impacts on activities of daily living that drive decision-making. Our study used established PGI-S and PGI-C items within qualitative interviews to better define the patient’s perspective of meaningful change in NSCLC and strengthen quantitative evaluations from PRO instruments. Assessing meaningful change in clinical trials is crucial to determine the clinical relevance of PROs (Ousmen et al., 2018). Anchor-based and distribution-based methods are commonly used. A recent publication quantitatively evaluated meaningful within-person change in advanced or metastatic NSCLC patients, using the NSCLC-SAQ and PGI-S as an anchor measure in the KEYNOTE-598 study, finding that the use of quantitative methods and focus on patients’ longitudinal perspectives of change while they received a specific treatment can limit applicability to the wider patient population (Williams et al., 2022). Combining qualitative insights (directly asking patients) with quantitative methods (indirect anchor- and distribution-based methods) in future studies would enable a more comprehensive understanding of meaningful change as anchor-based approaches can be used to assess differences cross-sectionally (e.g., between treatment arms) or longitudinally while distribution-based approaches are based on statistical measures (standard deviation of the scores, effect size and standard error of measurement) and do not require external criterion, potentially allowing findings to be compared to other studies using these methods (Ousmen et al., 2018). This integrated approach, recommended by FDA guidance workshops, enables a more accurate examination of quantitative data and better addresses the needs of patients within this population (Food and Drug Administration, 2018; Food and Drug Administration, 2019).

This study has several limitations, primarily related to recruitment. Firstly, our sample of 19 patients lacked diversity in both sex and race, and therefore may not be representative of the wider population of patients with NSCLC. Additionally, as this study only included patients from the US and those who were fluent in English, this further limits the generalizability of the findings to patients from other countries and cultural backgrounds. Further, despite recruiting for various disease stages, ultimately all 19 patients enrolled had Stage IV disease. However, the study was focused on the patient perspectives of those with advanced or metastatic NSCLC, so the qualitative data collected from patients with Stage IV disease was considered very important. Also, most patients had been diagnosed with NSCLC for >1 year (and 16% for >6 years), and therefore the perspectives of recently diagnosed patients may be underrepresented in our sample. Lastly, the majority of patients in our sample had been receiving some form of non-immunotherapy targeted therapy, frequently without any cytotoxic chemotherapy. This distribution of therapies may have led to an underrepresentation of the perspectives of patients receiving cytotoxic chemotherapy for advanced or metastatic NSCLC. Despite the limitations, our interview-based methodology allowed insight into the perspectives of patients with advanced or metastatic NSCLC and demonstrates the benefit of combining patient perspective interview-based instruments with core, quantitative PRO instruments. Some considerations for future studies include recruitment of a larger, more diverse patient population and across different countries. Surveys could be made available in multiple languages to enable non-English speakers to take part.

## 5. Conclusion

In this prospective interview-based qualitative research study, the most frequent spontaneously reported symptoms among patients with late-stage NSCLC were fatigue, shortness of breath/difficulty breathing, cough, pain, and GI issues. Many patients found it difficult to distinguish symptoms as either treatment-related or disease-related; however, all symptoms mentioned were reported to impact daily life, and it is therefore important to consider both treatment- and disease-related symptoms in future clinical trials. The PGI-S and PGI-C instruments were considered easy to understand and appropriate for use for patients with NSCLC. A one-point change on both PGI-S and PGI-C was deemed meaningful by most patients. We found asking patients to report meaningful change in terms of their own experience was a fruitful exercise when compared with traditional hypothetical scenarios. Our results also underlined the importance of considering both symptoms and daily life impacts, which may influence the selection of PRO instruments in clinical trials. Further, our interview-based methodology allowed insight into how meaningful change could be defined in terms of daily life impact, and therefore qualitative interviews may be a beneficial addition to quantitative PRO instruments.

## Data availability statement

GSK makes available anonymized individual patient data and associated documents from interventional clinical studies that evaluate medicines, upon approval of proposals submitted to https://www.gsk-studyregister.com/en/. To access data for other types of GSK sponsored research, for study documents without patient-level data, and for clinical studies not listed, please submit an enquiry via the website.

## Ethics statement

The study protocol was reviewed and approved by the WIRB-Copernicus Group, Inc. IRB (Review #: 20216504) before any study activities began. The study was conducted in accordance with the protocol, IRB requirements, applicable regulations and guidelines, and ethical principles for the conduct of research. The participants provided their written informed consent to participate in this study.

## Author contributions

AC, JH, and AS contributed to the concept and design of the study, data analysis, and interpretation. MS contributed the data analysis and interpretation. KK contributed towards the acquisition of data, data analysis, and interpretation. AM contributed towards the data analysis and interpretation. ARC contributed towards the acquisition of data, data analysis, and interpretation. ST contributed to the concept and design of the study. All authors contributed to the article and approved the submitted version.

## Conflict of interest

MS is an employee of GSK. JH is a former employee of GSK. AC and ST are employees of and hold stock in GSK. KK, AM, and ARC are employees of IQVIA. AS is a former employee of and holds stock in GSK, and is a employee of LumaBridge.

This study received funding from GSK (215353). The funder had the following involvement with the study: study design, collection (provided funding to the vendor IQVIA; GSK collaborated with IQVIA to determine how to collect the data but were not directly involved in patient recruitment or interviews), interpretation of data, the writing of this article and the decision to submit it for publication.

## Publisher’s note

All claims expressed in this article are solely those of the authors and do not necessarily represent those of their affiliated organizations, or those of the publisher, the editors and the reviewers. Any product that may be evaluated in this article, or claim that may be made by its manufacturer, is not guaranteed or endorsed by the publisher.
