# Supplementary material for: Perspectives of patients with advanced or metastatic non-small cell lung cancer on symptoms, impacts on daily activities, and thresholds for meaningful change: a qualitative research study
Source: Front Psychol. 2023 Sep 8;14:1217793. doi: 10.3389/fpsyg.2023.1217793 (PMC10516440; doi:10.3389/fpsyg.2023.1217793)
Supplement: Supplementary file 1 [file Table_1.docx]

# Supplementary Tables

## Supplementary Table 1. Patient-reported concept coverage in PRO instruments.

| **Concept** | **EORTC-QLQ-C30** | **EORTC-QLQ-LC13^†^** | **NSCLC-SAQ^†^** |
| --- | --- | --- | --- |
| SYMPTOMS | | | |
| Fatigue | Yes | No | Yes |
| Shortness of breath/difficulty breathing | Yes | Yes | Yes |
| Cough | No | Yes | Yes |
| Pain (e.g., chest, back) | Yes | Yes | Yes |
| GI issues (e.g., constipation, diarrhea, nausea)* | Yes | No | No |
| Dizziness* | No | No | No |
| Hair/skin/nail changes* | No | No | No |
| Changes in cognition | Yes | No | No |
| Changes in senses (e.g., vision, taste)* | No | No | No |
| Headache* | No | No | No |
| Lack of appetite | Yes | No | Yes |
| Physical weakness | Yes | No | No |
| Swelling | No | No | No |
| Wheezing | No | No | No |
| Symptom coverage, yes/total (%) | 7/14 (50) | 3/14 (21) | 5/14 (36) |
| IMPACTS | | | |
| Difficulty walking | Yes | N/A | N/A |
| Impact on social/interpersonal relationships | Yes | N/A | N/A |
| Sleep | Yes | N/A | N/A |
| Anxiety/depression | Yes | N/A | N/A |
| Difficulty doing daily tasks (e.g., showering, laundry) | Yes | N/A | N/A |
| Change in independence/ dependence | Yes | N/A | N/A |
| Exercise | No | N/A | N/A |
| Appreciation (e.g., for life, people, time) | No | N/A | N/A |
| Lack of motivation | No | N/A | N/A |
| Unable to work | Yes | N/A | N/A |
| Difficulty holding a conversation | No | N/A | N/A |
| Difficulty laughing | No | N/A | N/A |
| Travel | Yes | N/A | N/A |
| Change in weight (loss or gain) | No | N/A | N/A |
| Impact coverage, yes/total (%) | 8/14 (57) | 0 | 0 |
| Total concept coverage, yes/total (%) | 15/28 (54) | 3/28 (11) | 5/28 (18) |

*Symptoms described as treatment-related; †EORTC-QLQ-LC13 and NSCLC-SAQ measures are symptom scales and do not include evaluation of impacts. EORTC, European Organization for Research and Treatment of Cancer; GI, gastrointestinal; N/A, not applicable; NSCLC-SAQ, Non-Small Cell Lung Cancer Symptom Assessment Questionnaire; PRO, patient-reported outcomes; QLQ-C30, Core Quality-of-Life Questionnaire; QLQ-LC13, Quality-of-Life Questionnaire-Lung Cancer 13.
