# Supplementary material for: Perspectives of patients with advanced or metastatic non-small cell lung cancer on symptoms, impacts on daily activities, and thresholds for meaningful change: a qualitative research study
Source: Front Psychol. 2023 Sep 8;14:1217793. doi: 10.3389/fpsyg.2023.1217793 (PMC10516440; doi:10.3389/fpsyg.2023.1217793)
Supplement: Supplementary file 2 [file Table_2.docx]

# Supplementary Tables

## Supplementary Table 2. Time from diagnosis and meaningful change.

1. **PGI-S**

| **PGI-S scale** | **≤1 year since initial NSCLC diagnosis*, n (%) (N=4)** | **>1 year since initial NSCLC diagnosis*, n (%)**  **(N=15)** |
| --- | --- | --- |
| **Meaningful improvement** | | |
| 1-point change meaningful improvement | 2 (50) | 13 (87) |
| 2-point change meaningful improvement | 1 (25) | 1 (7) |
| **Meaningful worsening** | | |
| 1-point change meaningful worsening | 3 (75) | 13 (87) |
| 2-point change meaningful worsening | 0 | 2 (13) |

1. **PGI-C**

| **PGI-C measure** | **≤1 year since initial NSCLC diagnosis*, n (%) (N=4)** | **>1 year since initial NSCLC diagnosis*, n (%) (N=15)** |
| --- | --- | --- |
| **Meaningful improvement** | | |
| A little better: 1-point change meaningful improvement | 4 (100) | 13 (87) |
| Much better: 2-point change meaningful improvement | 0 | 2 (13) |
| **Meaningful worsening** | | |
| A little worse: 1-point change meaningful worsening | 3 (75) | 14 (93) |
| Much worse: 2-point change meaningful worsening | 1 (25) | 1 (7) |

*Please note that time since diagnosis may not represent time since diagnosis of Stage IV disease.
Two patients selected the “no symptom” response option for the last 7 days; therefore, no “improvement” response options were available for them when answering the PGI-S scale. One patient selected the “very severe” response option for the last 7 days; therefore, no “worsening” response options were available for them when answering the PGI-S scale. NSCLC, non-small cell lung cancer; PGI-C, Patient Global Impression of Change; PGI-S, Patient Global Impression of Severity.
